# Correlation between exposure (mAs) and image quality for a rapid cone‐beam CT on a ring gantry linear accelerator

**DOI:** 10.1002/acm2.70374

**Published:** 2025-11-18

**Authors:** Hui Zhao, Nicholas Nelson, Seth Streitmatter, Bradley Nordin, Thomas Martin, Ryan G. Price, Jeremy N. Kunz, Stephen Bhagroo, Y. Jessica Huang, Geoff Nelson

**Affiliations:** ^1^ Department of Radiation Oncology, Huntsman Cancer Hospital University of Utah Salt Lake City, Utah USA

**Keywords:** HyperSight CBCT, image quality, Rose criterion

## Abstract

**Background:**

The adoption of HyperSight CBCT has become widespread due to its rapid CBCT scan acquisition (< 6 s) and superior image quality. Numerous studies have been conducted to evaluate HyperSight CBCT's image quality and the accuracy of treatment planning dose calculations using HyperSight CBCT. Nevertheless, for the main function of CBCT as a tool of image guided radiation therapy (IGRT), one clinically significant question remains unanswered: What is the quantitative correlation between exposure level (mAs) and desired image quality? This study aims to explore the quantitative correlation between exposure level and image quality, providing guidelines for selecting the appropriate CBCT mAs based on imaging objectives and patient size for the optimal IGRT alignment.

**Purpose:**

To investigate the correlation between HyperSight CBCT (equipped on Halcyon linear accelerator) exposure level and image quality.

**Methods:**

Nine‐series of 261 sets CBCT scans were acquired using HyperSight on the Halcyon (125 kV, 133‐971 mAs, FOV: 28.2, 36, and 53.8 cm). Three‐series were acquired with the CatPhan 604 alone, three‐series with CatPhan 604 surrounded by a 32 cm custom‐made acrylic annulus, and three series with CatPhan 604 surrounded by a 40 cm custom‐made acrylic annulus. Image quality metrics were analyzed included signal‐to‐noise ratio (SNR), contrast‐to‐noise ratio (CNR), image noise, low‐contrast visibility (LCV), uniformity index (UI), integral non‐uniformity (INU), and modulation transfer function (MTF). The correlation between mAs and image quality was evaluated.

**Results:**

The SNR and CNR for all material inserts increased with rising mAs for all scans, with a higher amplitude of increase for scans without annuli. The SNR and CNR were higher for scans without annuli. Both image noise and LCV decreased with rising mAs and were higher for scans with annuli. The UI was independent of mAs for scans of CatPhan alone and CatPhan with a small annulus, and was slightly higher for scans without annuli (∼0.1 vs ∼0.2). The INU slightly increased with rising mAs and was higher for scans without annuli. MTF was independent of mAs and with or without annuli. MTF values varied with FOV, with smaller FOV yielding better image sharpness. All parameters were FOV‐dependent except INU, and generally, the larger the FOV, the better the image quality (except spatial resolution).

**Conclusions:**

HyperSight CBCT acquisition is recommended to be performed with a maximum FOV of 53.8 cm. To meet the Rose criterion, for a 20 cm width object, CBCT should be acquired at ≥154 mAs; for a 32 cm width object, CBCT should be acquired at ≥572.6 mAs to reach a 3σ confidence level; for a 40 cm object, CBCT should be acquired at ≥572.6 mAs to reach a 3σ confidence level for all materials except polystyrene (soft‐tissue).

## 1 INTRODUCTION

Since the initial clinical implementation of HyperSight CBCT system (Varian Medical Systems, Palo Alto, CA) on Halcyon in February 2023, its adoption has become widespread. This is largely due to its rapid CBCT scan acquisition (<6 s) and superior image quality, comparable to that of simulation CT. Numerous studies have been conducted to evaluate HyperSight CBCT's image quality^1^, ^2^, ^3^, ^4^, ^5^, ^6^, ^7^, ^8^, ^9^ and the accuracy of treatment planning dose calculations using HyperSight CBCT.^10^, ^11^, ^12^, ^13^ Nevertheless, for the main function of CBCT as a tool of image guided radiation therapy (IGRT), one clinically significant question remains unanswered: What is the quantitative correlation between exposure level (mAs) and desired image quality?

Before acquiring a CBCT scan, the appropriate protocol is typically selected based on the treatment site. Varian's Halcyon system offers a range of mAs values for each CBCT protocol, allowing users to tailor image quality to clinical needs. However, selecting the optimal mAs for individual patients remains a challenge, particularly due to patient size variability, which significantly affects image quality through changes in scatter, noise, and ultimately IGRT accuracy.

Optimizing CBCT imaging requires careful consideration of multiple factors, including patient size, image quality requirements, and radiation dose—each of which plays a critical role in clinical decision‐making. Previous studies have explored protocol optimization for conventional CBCT systems, emphasizing the need for patient‐specific approaches due to the impact of body size on scatter, noise, and IGRT accuracy.^14^, ^15^

This study builds on that foundation by quantitatively evaluating the correlation between exposure level (mAs) and image quality using annulus phantoms in the context of HyperSight CBCT. The goal is to provide practical guidelines for selecting appropriate mAs settings based on imaging objectives and patient size. These findings support the development of more personalized and effective IGRT alignment strategies, while also contributing to dose‐aware imaging practices.

## 2 MATERIALS AND METHODS

All CBCT scans were acquired at 125 kVp, representing the most commonly used energy setting in clinical practice. Three fields of view (FOV) were utilized for HyperSight CBCT scans: 28.2, 36, and 53.8 cm. For each FOV, 29 CBCT scans were acquired using varying mAs values (133–971). In clinical mode on Halcyon, for CBCT acquired at 125 kVp, a range of non‐continuous mAs values between 132.93 and 971.38 was available for selection. The 29 specific mAs values used in this study were chosen with intervals of 20–49 mAs. The reconstruction method employed was iCBCT Acuros, which improves soft‐tissue contrast, image uniformity, and HU accuracy by implementing further scatter correction compared to iCBCT alone. A slice thickness of 3 mm was automatically reconstructed using the HyperSight iCBCT Acuros pelvis protocol for all scans performed in clinical mode. Each CBCT scan was acquired in 5.9 s using a single full‐fan selection and a half‐gantry rotation of 211°.

**FIGURE 1:**
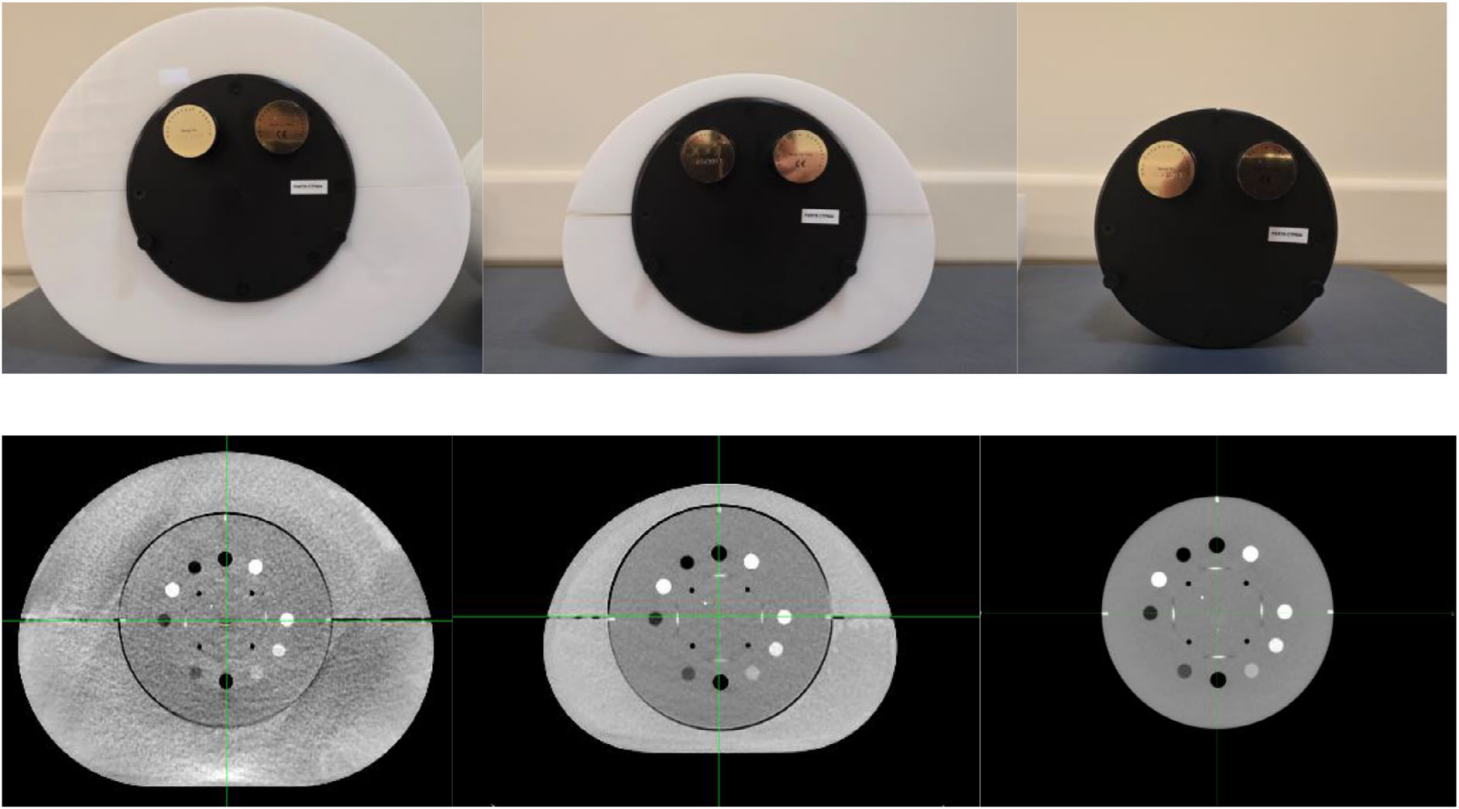
Top: CBCT scan setups showcasing the 40 cm annulus surrounding the CatPhan, the 32 cm annulus surrounding the CatPhan, and the CatPhan alone. Bottom: Axial slices corresponding to each CBCT scan setup.

For each FOV, scans were acquired on a CatPhan 604 (The Phantom Laboratory, Inc., Greenwich, NY), which has a diameter of 20 cm. Two custom‐made acrylic annuli, measuring 32 and 40 cm widths, were used to surround the CatPhan. The top portion of Figure 1 illustrates the setup: the CatPhan alone, the CatPhan with the 32 cm annulus, and the CatPhan with the 40 cm annulus. The CatPhan with the 32 cm annulus may represent the pelvis for a medium size patient, and the CatPhan with the 40 cm annulus may represent the pelvis for a large size patient. The bottom portion of Figure 1 presents the corresponding axial slices of the CBCT scans.

In total, 261 CBCT scans were acquired. Image quality metrics were analyzed for all scans, including signal‐to‐noise ratio (SNR), contrast‐to‐noise ratio (CNR), image noise, low‐contrast visibility (LCV), uniformity index (UI), integral nonuniformity (INU), and modulation transfer function (MTF) for spatial resolution. Additionally, contouring and relative mean Hounsfield unit (HU) calculations were performed in MIM (MIM Software Inc., Cleveland, OH) to assess image quality.

Image noise was defined as the standard deviation of pixel values within homogenous regions of interest (ROI). In this study, noise was calculated with an average of five ROIs located at the center, 12 o'clock, 3 o'clock, 6 o'clock, and 9 o'clock positions, following the guideline of AAPM TG‐233^16^. Each ROI had a diameter of 2 cm. Image noise was quantified by reporting the standard deviation as a percentage of the mean pixel value, averaged across all ROIs^17^.

The SNR was calculated using Equation 1, where the $\mu_{insert}$ represents the mean HU number of a specific insert, and $\sigma_{BG}$ is the standard deviation of the background. The mean HU number of a specific insert was determined within a ROI contoured as a cylindrical structure with a diameter of 80% of the insert and a length of 2.2 cm. The background ROI was contoured with a diameter of 3 cm and a length of 2.2 cm.

(1) $$SNR=\frac{\left|\mu_{\mathrm{insert}}\right|}{\sigma_{\mathrm{BG}}}$$

The CNR represents the difference in signal intensity between two regions, scaled to the image noise. CNR was calculated using Equation 2,^18^ where $\mu_{insert}$ and $\mu_{BG}$ were the mean HU number of a specific insert and background, respectively. $\sigma_{insert}$ and $\sigma_{BG}$ were the standard deviations of HU numbers of the insert and the background.

(2) $$CNR=\frac{\left|\mu_{\mathrm{insert}}-\mu_{\mathrm{BG}}\right|}{\sigma_{\mathrm{BG}}}$$

The LCV refers to the ability to distinguish between materials with similar HU numbers. In this study, polystyrene (HU range provided by CatPhan: –65 to –29) and low‐density polyethylene (LDPE; HU range: –121 to –87) were chosen to evaluate LCV. LCV was calculated using Equation 3,^19^ where $\mu_{\mathrm{polystyrene}}$ and $\mu_{\mathrm{LDPE}}$ were the mean pixel values of the polystyrene and the LDPE inserts. $\sigma_{\mathrm{polystyrene}}$ and $\sigma_{\mathrm{LDPE}}$ were the standard deviations of the pixel values of the polystyrene and the LDPE inserts.

(3) $$LCV=2.75\times \frac{\sigma_{\mathrm{polystyrene}}+\sigma_{\mathrm{LDPE}}}{\mu_{\mathrm{polystyrene}}-\mu_{\mathrm{LDPE}}}$$

UI is used to demonstrate image uniformity, which refers to the consistency of HU numbers across a homogenous region of the image. UI was calculated using Equation 4, where $\mu_{\mathrm{periphery}}$ was the HU number of the periphery and $\mu_{\mathrm{center}}$ was the mean HU number of the center ROIs.

(4) $$UI=\mu_{\mathrm{periphery}}-\mu_{\mathrm{center}}$$

Image INU refers to the variation in image intensity and represents the maximum deviation from the mean intensity. INU was calculated using Equation 5, where $\mu_{\max}$ was the maximum mean pixel value of all ROIs, and $\mu_{\min}$ was the minimum mean pixel value of all ROIs.

(5) $$INU=\frac{\mu_{\max}-\mu_{\min}}{\mu_{\max}+\mu_{\min}}$$

The relative MTF (rMTF) was calculated using the open‐source software **pylinac** (https://pylinac.readthedocs.io/en/latest/cbct.html). Fifteen ROIs were drawn, each corresponding to bar patterns with fixed spatial frequencies ranging from 1 to 15 line pairs per millimeter (lp/mm), in 1 lp/mm increments. For each ROI/bar pattern, contrast was calculated using Equation 6, where $I_{\max}$ and $I_{\min}$ represent the maximum and minimum pixel intensities within the ROI. The rMTF was then computed using Equation 7 and tabulated as a function of lp/mm. MTF values were assessed at contrast levels of 1%, 2%, 5%, 10%, 20%, 30%, 40%, 50%, 60%, 70% 80%, 90%, 95%, and 100%, enabling a relative comparison of MTF across all evaluated CBCT scans.

(6) $$I=\frac{I_{\max}-I_{\min}}{I_{\max}+I_{\min}}$$

(7) $$rMTF=\frac{\left(I_{ROI,\;i}\right)}{\left(I_{ROI,\;1\;lp/mm}\right)}$$

## 3 RESULTS

### 3.1 SNR and CNR

**FIGURE 2:**
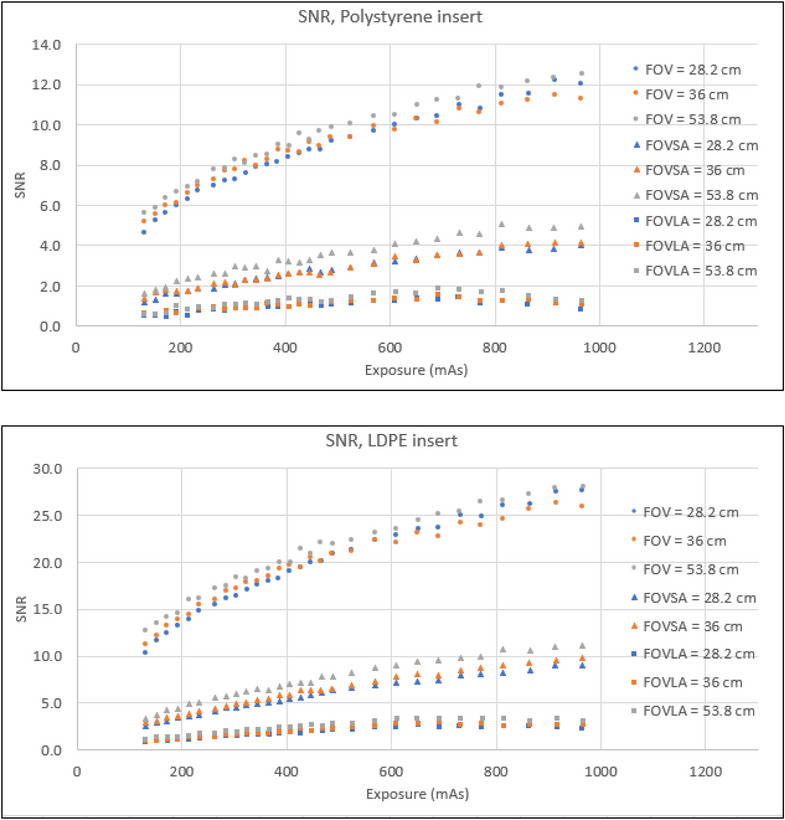
SNR for polystyrene and LDPE inserts.

**FIGURE 3:**
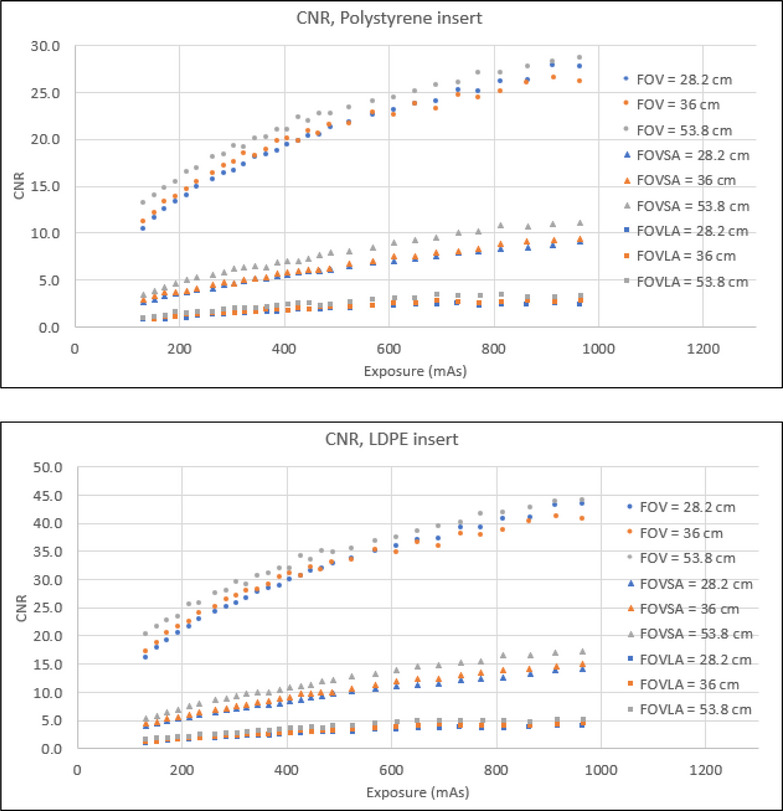
CNR for polystyrene and LDPE inserts.

Table 1 summarizes the range of SNR and CNR of all mAs across all material inserts, respectively. Four key findings emerged from the SNR and CNR evaluation. First, both SNR and CNR generally increased with the increase of mAs for all scans and all material inserts. However, for the LDPE insert, scans of CatPhan with a large annulus showed the SNR plateau beyond 700mAs. For the polystyrene insert, scans of CatPhan with a large annulus exhibited minimal SNR variation (0.6 to 1.3 for FOV 53.8 cm) across all mAs. For the polystyrene insert, scans of CatPhan with a large annulus showed minimal CNR variation (1.1 to 3.4 for FOV 53.8 cm). Second, the amplitude of both SNR and CNR increase with rising mAs was highest for scans of CatPhan alone and lowest for scans of CatPhan with a large annulus. Third, the SNR and the CNR for scans of CatPhan with a small annulus were reduced by roughly a factor of 3 compared to scans of CatPhan alone at the same mAs. The SNR and the CNR for scans of CatPhan with a large annulus were further reduced by approximately another factor of 3 compared to scans of CatPhan with a small annulus. Fourth, the highest SNR and CNR were observed for an FOV of 53.8 cm. Below 500mAs, the SNR and the CNR for an FOV of 36 cm were higher than for an FOV of 28.2 cm. However, beyond 500mAs, the SNR and the CNR for an FOV of 36 cm were lower than for an FOV of 28.2 cm. Figure 2 and Figure 3 illustrate the correlation between SNR/CNR and mAs for polystyrene and LDPE inserts across all FOVs, with and without small and large annuli.

**TABLE 1:** The range of SNR and CNR of all mAs for all material inserts.

**SNR**									
Material	FOV 28.2 cm			FOV 36 cm			FOV 53.8 cm		
	NoA*	SA*	LA*	NoA	SA	LA	NoA	SA	LA
Air	103.6–272.9	26.7–90.7	7.8–28.7	108.7–257.6	28.6–96.1	8.5–30.3	126.1–275.9	34.0–110.8	10.4–35.9
PMP*	20.1–52.8	5.2–17.5	1.4–4.8	21.2–50.3	5.4–18.5	1.5–5.2	24.4–54.0	6.7–21.6	1.9–6.4
LDPE*	10.3–27.7	2.6–9.1	0.9–2.4	11.2–26.3	2.9–9.8	1.0–2.8	12.7–28.1	3.4–11.1	1.2–3.2
Polystyrene	4.6–12.2	1.2–4.0	0.6–1.1	5.2–11.5	1.4–4.1	0.7–1.1	5.6–12.5	1.6–4.9	0.6–1.3
Acrylic	11.8–32.1	2.9–10.6	0.6–3.8	12.6–30.8	3.5–11.6	0.8–4.3	15.1–33.1	3.7–13.3	1.1–4.8
Delrin	35.5–94.3	9.1–31.4	2.2–9.7	37.4–89.4	9.7–33.5	2.4–10.1	43.8–95.9	11.2–38.8	3.1–11.7
Hydroxyapatite 50%	70.0–184.4	17.1–58.2	4.4–17.6	73.8–175.1	18.1–62.2	4.8–18.6	85.8–187.8	22.2–71.8	6.1–21.8
Hydroxyapatite 20%	23.3–62.0	5.3–19.2	1.3–6.4	24.4–58.7	5.9–20.3	1.3–6.6	28.9–63.1	7.2–23.8	1.9–7.8
Teflon	97.9–258.9	25.5–86.2	7.0–26.9	102.7–244.6	26.8–90.9	7.6–28.4	120.0–264.0	31.7–106.4	9.6–33.3
**CNR**									
material	FOV 28.2 cm			FOV 36 cm			FOV 53.8 cm		
	NoA	SA	LA	NoA	SA	LA	NoA	SA	LA
Air	109.4–288.5	28.1–95.7	8.0–30.4	114.9–272.7	30.2–101.4	8.8–32.1	133.7–292	35.9–117.0	10.9–37.9
PMP*	25.9–68.5	6.6–22.6	1.6–6.5	27.4–65.3	7.0–23.8	1.8–7.0	32.0–70.1	8.5–27.8	2.3–8.4
LDPE	16.1–43.5	4.0–14.1	1.1–4.1	17.3–41.3	4.5–15.1	1.3–4.5	20.2–44.2	5.3–17.3	1.7–5.2
Polystyrene	10.5–27.9	2.6–9.1	0.8–2.6	11.3–26.5	2.9–9.5	1.0–2.9	13.2–28.6	3.5–11.1	1.1–3.4
Acrylic	6.0–16.4	1.5–5.6	0.4–2.2	6.4–15.8	1.9–6.3	0.4–2.6	7.5–17.0	1.8–7.1	0.6–2.7
Delrin	29.7–78.6	7.6–26.4	1.9–8.0	31.3–74.4	8.2–28.2	2.1–8.3	36.2–79.8	9.4–32.6	2.7–9.8
Hydroxyapatite 50%	64.2–168.6	15.7–53.2	4.1–16.0	67.6–160.1	16.6–56.9	4.5–16.8	78.2–171.1	20.3–65.7	5.6–19.7
Hydroxyapatite 20%	17.4–46.3	3.9–14.2	1.0–4.8	18.3–43.6	4.4–15.0	1.0–4.8	21.3–47.0	5.4–17.6	1.4–5.7
Teflon	92.1–243.2	24.0–81.1	6.8–25.2	96.6–229.5	25.2–85.6	7.3–26.6	112.4–247.9	29.8–100.2	9.1–31.3

*PMP, polymethylpentene; *LDPE, low–density polyethylene; *NoA, no annulus; *SA, small annulus; *LA, large annulus.

### 3.2 Image noise

**FIGURE 4:**
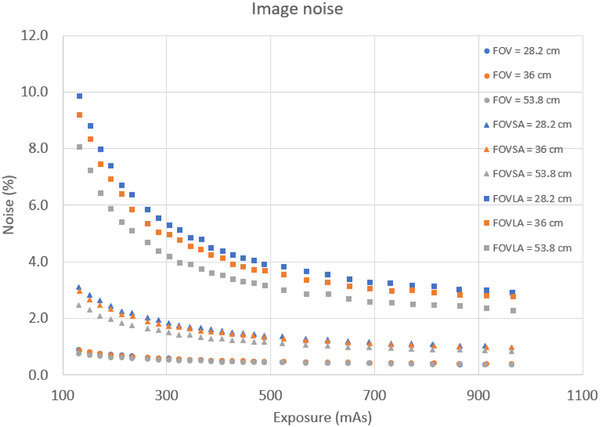
Imaging noise for all CBCT scans.

Four key findings emerged from the image noise evaluation. First, image noise decreased with the increase of mAs for all scans. Second, the amplitude of the image noise decrease was highest for scans of CatPhan with a large annulus, while scans of CatPhan alone showed only about 0.5% change in image noise. Third, image noise for scans of CatPhan with a small annulus increased by roughly a factor of 3 compared to scans of CatPhan alone. For scans of CatPhan with a large annulus, image noise increased by approximately another factor of 3 compared to scans of CatPhan with a small annulus. Fourth, image noise was lowest for an FOV of 53.8 cm, and highest for an FOV 28.2 cm. Figure 4 shows image noise plot for all CBCT scans.

### 3.3 LCV

**FIGURE 5:**
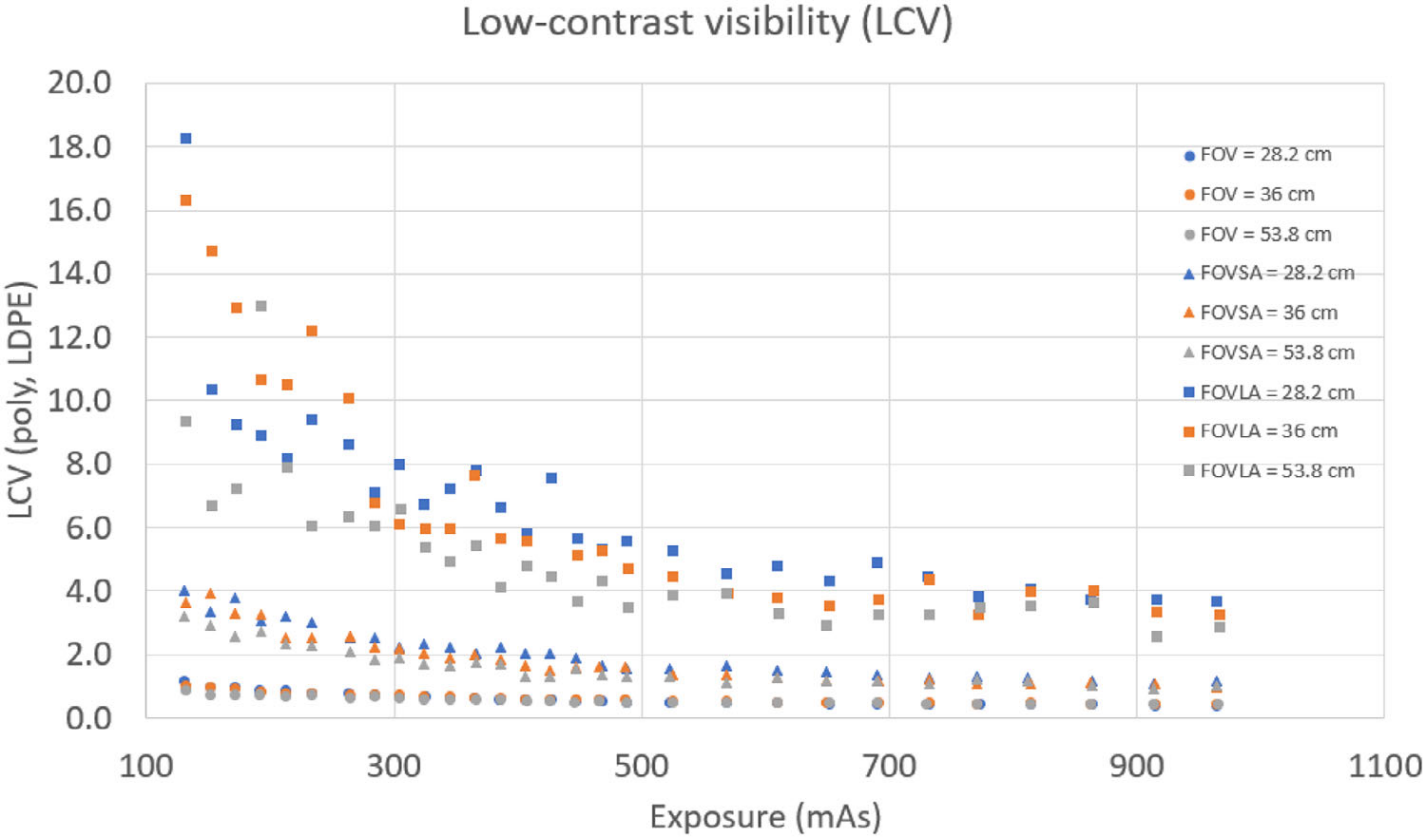
LCV (polystyrene and LDPE) for all CBCT scans.

Four key findings emerged from the LCV evaluation. First, LCV generally decreased with the increase of mAs for all scans. Second, the amplitude of the LCV decrease with rising mAs was the highest for scans of CatPhan with a large annulus, while scans of CatPhan alone showed a slight decrease in LCV (0.4–0.9 for FOV 53.8 cm). Third, LCV for scans of CatPhan with a small annulus increased by roughly a factor of 3 compared to scans of CatPhan alone. For scans of CatPhan with a large annulus, LCV showed significant variation for mAs less than 500. Fourth, LCV was the lowest for an FOV of 53.8 cm and the highest for an FOV 28.2 cm. Figure 5 showed the LCV plot for all CBCT scans.

### 3.4 UI

**FIGURE 6:**
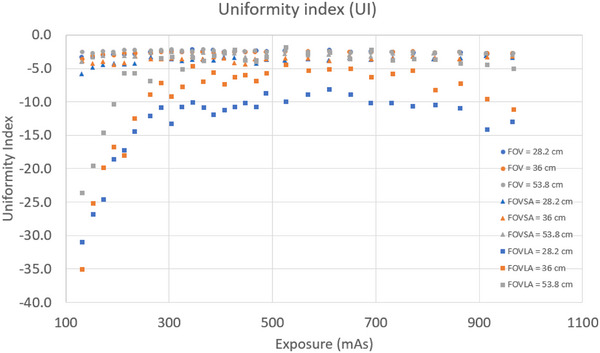
UI for all CBCT scans.

Three key findings emerged from the UI evaluation. First, UI was generally independent of mAs for scans of CatPhan alone and CatPhan with a small annulus. However, for scans of CatPhan with a large annulus, UI increased significantly with rising mAs at mAs < 300, and was independent of mAs at mAs > 300. Second, UI for scans of CatPhan with a small annulus decreased slightly compared to scans of CatPhan alone (–2.9 vs. –2.7 for FOV 53.8 cm, 971.38 mAs). UI for scans of CatPhan with a large annulus showed a slight decrease compared to scans of CatPhan with a small annulus at mAs > 300 (–5.0 vs. –2.9 for FOV 53.8 cm, 971.38 mAs). Third, UI was lowest for an FOV of 53.8 cm and highest for an FOV 28.2 cm. Figure 6 showed the UI plot for all CBCT scans.

### 3.5 INU

**FIGURE 7:**
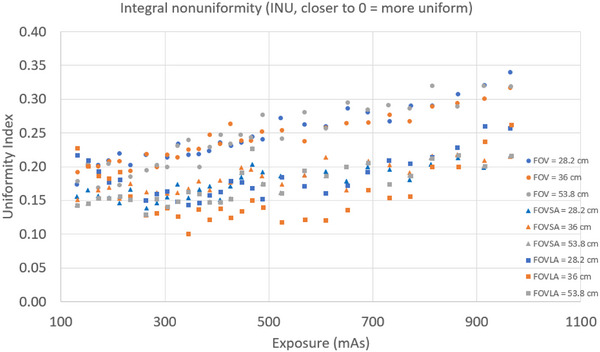
INU for all CBCT scans.

Three key findings emerged from the INU evaluation. First, INU showed a slight trend of increase with rising mAs for all scans. Second, INU for scans of CatPhan with small and large annuli decreased slightly compared to scans of CatPhan alone. Third, INU had no clear correlation with FOV. Figure 7 showed the INU plot for all CBCT scans.

### 3.6 MTF

**FIGURE 8:**
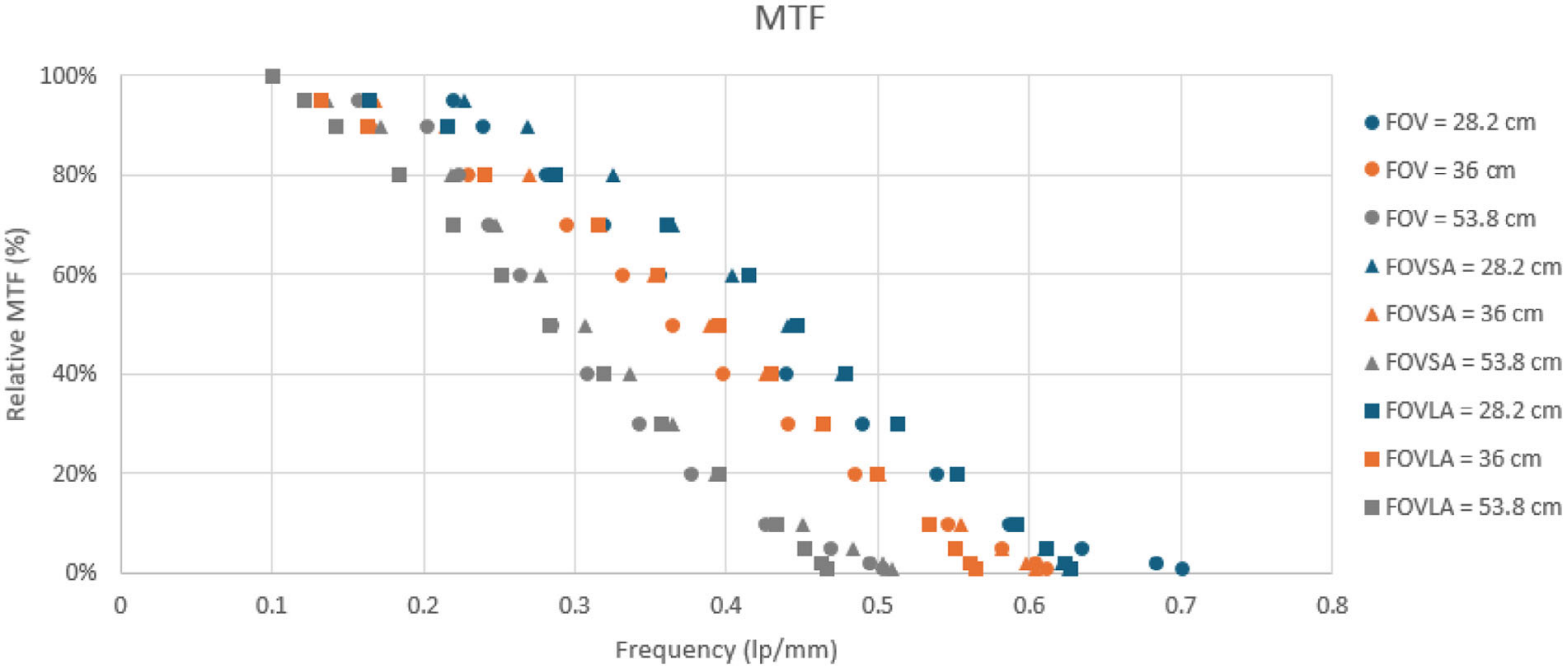
MTF curves for scans conducted at 971.38 mAs.

Three key findings emerged from the MTF evaluation. First, MTF was found to be independent of mAs. Second, slight variations in MTF were observed between scans performed with and without annuli. Third, MTF values varied with FOV: the lowest MTF was recorded at an FOV of 53.8 cm, while the highest was observed at 28.2 cm. These trends are illustrated in Figure 8, which presents the MTF curves for scans conducted at 971.38 mAs.

## 4 DISCUSSION

There are four available kVp settings for HyperSight CBCT: 80, 100, 125, and 140 kVp. Among these, 125 kVp is the most commonly used in clinical practice and is one of the two energies (125 and 140 kVp) supported by Varian for treatment planning protocols (iCBCT Acuros and iCBCT MAR). Although other kVp values are available, our study focused on 125 kVp to reflect standard clinical usage and to establish a general relationship between mAs and image quality. This relationship is largely independent of kVp, as the trends observed are consistent across different energy levels. The slice thickness of 3 mm was selected inadvertently. The HyperSight iCBCT Acuros pelvis protocol offers two slice thickness options: 2 and 3 mm. Although we acknowledge that a 2 mm slice thickness is ideal for higher spatial resolution, the 3 mm slice thickness remains valid for the image quality metrics evaluated in this study due to the averaging nature of the calculation algorithms used. For treatment planning purposes, Varian provides a dedicated HyperSight CBCT acquisition protocol with a slice thickness of 1.5 mm, specifically designed to support high‐resolution imaging needs in adaptive workflows.

In this study, CBCT scans acquired with the largest FOV of 53.8 cm showed slightly better SNR, CNR, image noise, and LCV. The larger FOV corresponds to the larger image voxels, which reduce image noise. Improved SNR, CNR, and LCV are the results of this reduced image noise. Although spatial resolution decreases slightly with increasing FOV, this reduction is not considered to significantly impact the IGRT process compared to other factors such as SNR, CNR, and image noise. Based on the results from this study, an FOV of 53.8 cm is recommended for all patient CBCT scans using HyperSight.

Previous investigations have verified the plausibility of implementing the Rose criterion in CT imaging, which states that an SNR of at least 5 is needed to be able to distinguish image features^20^, ^21^ A SNR of 3 (3σ) is the minimally acceptable with a confident level of 99.7%. In our study, for scans of CatPhan alone (20 cm diameter), the SNR was > 5 at ≥153.88 mAs for all inserts. For scans of CatPhan with a small annulus (32 cm width), the SNR was over 5 for all materials at exposure ≥368.1 mAs except polystyrene. For polystyrene, the SNR was less than 5 even at the maximum mAs. To reach the minimal acceptable SNR of 3, the exposure was needed to be at 572.6 mAs. For scans of CatPhan with a large annulus (40 cm width), the SNR was less than 5 for at least three material inserts even at the maximum mAs. To reach the minimal acceptable SNR of 3, the exposure was needed to be at 572.6 mAs for all materials except polystyrene. The highest SNR for polystyrene was 1.3 (at maximum mAs for an FOV of 53.8 cm). In summary, to meet the Rose criterion, CBCT needs to be acquired at ≥153.88 mAs for a 20 cm width object to achieve SNR of 5. For 32 and 40 cm width objects, CBCT needs to be acquired at ≥572.6 mAs to reach the 3σ confidence level. However, for the 40 cm object, polystyrene cannot achieve the minimum SNR even at maximum exposure level.

Varian's Halcyon system features an auto‐mAs function that calculates the required mAs based on patient size obtained from simulation CT. The auto‐mAs for the CatPhan alone was 163.6, while for the CatPhan with a small annulus, it was 613.5. For the CatPhan with a large annulus, the auto‐mAs was set to the maximum (971.38) accompanied by a message indicating the limitations of the current kV and mAs settings. When the kV was increased to 140, the auto‐mAs reached the maximum of 1489.63, with the same message about the limitations of current settings. In our study, the required mAs was 5.9% and 6.7% lower than the auto‐mAs provided by Varian for the CatPhan alone (153.88 vs 163.6) and the CatPhan with a small annulus (572.6 vs 613.5), respectively. For the CatPhan with a large annulus, no mAs within the range could achieve the desired image quality, according to both our study and the Varian auto‐mAs.

SNR, CNR, and image noise were reduced by roughly a factor of 3 compared scans of CatPhan with a small annulus to CatPhan alone, and by approximately another factor of 3 compared scans of CatPhan with a large annulus to CatPhan with a small annulus. For scans of CatPhan with a large annulus (object side 40 cm) at maximum mAs (971.38), the SNR for polystyrene was 1.3 and the CNR was 3.4. Both SNR and CNR for polystyrene remained fairly consistent at ≥700 mAs. Polystyrene has a similar HU number to soft‐tissue; therefore, for a patient body size of 40 cm, soft‐tissue visibility would be limited even at the maximum mAs.

The observed plateaus in SNR and CNR values at ≥700 mAs levels are likely due to the dominance of scatter and the saturation of signal improvement. As mAs increases, the primary signal improves initially, leading to better contrast and reduced noise. However, beyond a certain point, the contribution of scatter becomes more significant, limiting further gains in image quality. This results in a plateau where additional mAs no longer yield proportional improvements in SNR or CNR. This behavior is consistent with known CBCT imaging physics and highlights the importance of identifying optimal mAs thresholds to avoid unnecessary dose without compromising image quality.

A smaller LCV number indicates better low‐contrast visibility. The LCV changed minimally at mAs ≥ 500 for all scans. Even for scans of CatPhan with a large annulus, the reduction of LCV for an FOV of 53.8 cm was about 1 beyond 500mAs.

Both scans with CatPhan alone and CatPhan with a small annulus showed similar UI and independence between UI and mAs. However, the UI for scans of CatPhan with a large annulus varied with rising mAs. The UI increased significantly with rising mAs at mAs < 300, and was independent of mAs at mAs > 300. This is because the amplitude of the image noise decrease was higher when the mAs < 300. The relatively stable INU showed small variation in image intensity for all scans.

The finding that MTF is independent of mAs suggests that variations in exposure do not significantly impact image sharpness. Minor difference in MTF between scans performed with and without annuli indicate a limited influence of structural elements on image sharpness. Additionally, MTF values varied with FOV, with smaller FOVs yielding better image sharpness.

Although this study was conducted using annulus phantoms to simulate varying patient sizes, the methodology and results are applicable to a wide range of anatomical sites and clinical scenarios. The quantitative relationship between mAs and image quality metrics such as SNR, CNR, and LCV can inform protocol selection not only for pelvic imaging but also for other treatment sites where soft‐tissue visualization is critical. Furthermore, the mAs guidelines derived from this study can be adapted for use in real patient imaging, supporting personalized IGRT workflows. By accounting for patient size and imaging objectives, these recommendations can help optimize image quality while minimizing radiation dose. This approach aligns with current trends in patient‐specific imaging and has the potential to inform vendor algorithm development for automated protocol selection in clinical practice.

In our study, across all mAs, the HU variation for all scans was within ± 50HU for bone and ± 20HU for other material inserts regardless of the phantom dimension and FOV settings. According to Davis et al^22^ a range of HU tolerances of 40HU for soft tissue and 100HU for the lung and bone would restrict dose changes to <1% in the treatment plan.

This study focuses on characterizing the correlation between mAs and image quality in HyperSight CBCT. Our previous work comparing image quality between HyperSight and traditional CBCT has been published.^1^ Currently, we are conducting a follow‐up study comparing image quality between Varian TrueBeam and HyperSight CBCT using the same image quality metrics applied in this investigation.

## 5 CONCLUSIONS

Acquiring CBCT scans with appropriate mAs settings is essential for accurately distinguishing soft‐tissue boundaries and achieving reliable IGRT alignment. Based on our findings, HyperSight CBCT acquisition is recommended to be performed with a maximum field of view (FOV) of 53.8 cm to optimize image quality metrics such as SNR, CNR, image noise, and LCV. To meet the Rose criterion for detectability, for a 20 cm width object, a minimum of 154 mAs is required to achieve an SNR of 5; for a 32 cm object, at least 572.6 mAs is needed to reach a 3σ confidence level; for a 40 cm object, 572.6 mAs is also necessary to meet a 3σ threshold across all materials, though soft‐tissue visibility remains a limited factor. These results provide actionable mAs guidelines that can support the development of personalized IGRT protocols, reduce unnecessary imaging dose, and potentially inform vendor algorithms for automated parameter selection—contributing to broader clinical impact and improved patient care.

## AUTHOR CONTRIBUTIONS

All authors made direct intellectual contributions to the work

## CONFLICT OF INTEREST STATEMENT

The authors declare no conflict of interest.
